# Separation of oil vapour by polyether block amide composite membrane modified with porous materials

**DOI:** 10.1098/rsos.220008

**Published:** 2022-11-23

**Authors:** Jiangyi Yan, Yuting Cai, Rong Xu, Beifu Wang, Lihong Nie

**Affiliations:** ^1^ College of Petrochemical Engineering and Environment, Zhejiang Ocean University, No.1 Haida South Road, Lincheng Street, Dinghai District, Zhoushan, Zhejiang 316000, People's Republic of China; ^2^ College of Naval Architecture and Maritime, Zhejiang Ocean University, No.1 Haida South Road, Lincheng Street, Dinghai District, Zhoushan, Zhejiang 316000, People's Republic of China

**Keywords:** microporous zeolite, mesoporous zeolite, polyether block amide, composite membrane, gas separation, oil vapour

## Abstract

The ability of membranes to separate oil vapour is affected by their permeance and selectivity. This study modifies polyether block amide (PEBA) composite membranes with a microporous zeolite, Silicalite-1, or a mesoporous zeolite, MCM-41. The results show that when PEBA composite membranes are modified with these zeolites, the selective layer of the composite membrane is coated more thinly, resulting in a higher flux of organic gas. Silicalite-1 increases the hydrophobicity of the membrane, which facilitates the adsorption of organic vapour on the membrane surface, thus improving the membrane selectivity. In the separation of oil vapour, both modified membranes can effectively increase the gas permeabilities and selectivities. The main mechanism governing gas transport in the MCM-41-modified membrane is Knudsen diffusion, so the selectivity for small molecules is improved more significantly. By contrast, the dissolution–diffusion mechanism is dominant in the Silicalite-1-modified membranes, which considerably increases the selectivity for large molecules.

## 1. Introduction

Atmospheric pollution is one of the most prominent environmental problems in the world, and oil vapour emissions from petrochemical enterprises are an important source of air pollutants [1–3]. Recycling oil vapour can not only avoid the loss of petroleum and save energy but also improve the air quality of the operating environment and effectively reduce environmental pollution [4–6]. Oil vapour recovery technologies include membrane separation [7], condensation [8,9], adsorption [10–12] and absorption [13,14]. Among them, membrane separation technology has several important advantages, such as flexible and simple operation, reliable performance, high recovery rates and no secondary pollution [15,16]. Polymeric membranes for oil vapour separation have been extensively prepared from various materials such as polydimethylsiloxane [17], polyamide [18], polyimide [19], polyether block amide (PEBA) [20] and polysulfone [21]. However, these conventional polymers involve a difficult trade-off between gas permeability and separation selectivity; thus, they must be modified, whether by processing methods or other materials.

Inorganic porous materials consist of interpenetrating or closed pores. They possess the advantages of a high specific surface area, an empty skeleton structure and abundant holes [22]. In recent years, because of their outstanding performance in separation, adsorption and catalysis, they have been widely adopted in energy, science, architecture, environmental sustainability and other fields [23,24]. The function of porous materials is affected not only by the pore size but also by the pore structure and other characteristics [25]. MCM-41 is a representative mesoporous zeolite. Because of its unique uniform hexagonal mesoporous structure, its good pore size distribution and its straight and unconnected channels [26], it has attracted considerable attention as an adsorbent, a catalyst carrier and even a hydrogenation catalyst. Silicalite-1 is a pure silica Mordenite framework inverted (MFI)-type zeolite molecular sieve. Because its silicon-to-aluminium ratio tends to infinity, the skeleton is electrically neutral and does not have a cation exchange capability. The micropores in its Si–O–Si structure act as adsorption sites and have outstanding advantages in areas such as membrane separation and adsorption separation [27].

Recent research has focused on hybrid matrix membranes, and different combinations of matrix materials and filler compositions have been investigated [28,29]. Thus far, MCM-41 has been applied to prepare hybrid matrix membranes based only on conventional polymers, such as polysulfone membranes [30–32], polyethylene membranes [33,34] or copolyimide membranes based on 6FDA [35]. Notably, the permeability of the MCM-41-zeolite-modified membranes for gas separation usually increases without a loss of selectivity. In gas separation applications, two main diffusion mechanisms occur, namely, Knudsen diffusion and dissolution–diffusion. Gas is usually transported through the polymer matrix through the dissolution–diffusion mechanism, which depends on the ability of gas molecules to dissolve in the polymer surface layer. Meanwhile, gas transport through pores inside the filler particles usually occurs via Knudsen diffusion. This transport mechanism can also be activated when interfacial pores exist between filler particles and the polymer matrix. Therefore, the size of these molecular space or pores is crucial for determining the type of the gas that will be separated as well as the amount [36].

This study aimed to investigate the effects of different zeolites on the membrane structure and performance and to study the influence of the zeolite pore size on the separation of oil vapour. To that end, PEBA/MCM-41 and PEBA/Silicalite-1 composite membranes were prepared using MCM-41 zeolite and Silicalite-1 zeolite as modifying materials. To the best of our knowledge, this is the first study on the effect of the different pore sizes of molecular sieves on gas separation performance. Through this research, we have gained a better understanding of the modes of gas transport and separation effects of molecular sieve-modified gas separation membranes.

## 2. Material and methods

### 2.1. Materials

PEBA was purchased from Aladdin Industrial Corporation (USA). Polyether sulfone (PES) was obtained from Solvay (USA). MCM-41 and Silicalite-1 zeolites were obtained from Zhuoran Environmental Protection Technology (Dalian) Co., Ltd to modify the polymeric membrane materials. Polyvinylpyrrolidone (PVP) supplied by BASF (Germany) was used as the pore-foaming agent. N-methyl pyrrolidone (NMP, 99.9%) and n-butanol were provided by Sinopharm Chemical Reagent Co., Ltd. Deionized water served as the non-solvent.

### 2.2. Preparation of composite membranes

To fabricate the support layer, PES (52.5 g) and PVP (29.17 g) were dissolved in NMP (210 g) under gentle stirring for 48 h at 90°C. The resulting casting solution was held in a vacuum oven at 60°C for 12 h to remove bubbles. Then, a flat membrane with a thickness of 50 µm was prepared using a coating blade (Guangzhou DMY Instrument Co.,Ltd, China) and immediately placed in a prepared coagulation bath. After the membrane phase converted from liquid to solid, it was washed thoroughly with deionized water to eliminate chemical residue. Finally, the support layer was dried for subsequent testing.

**Figure 1.**
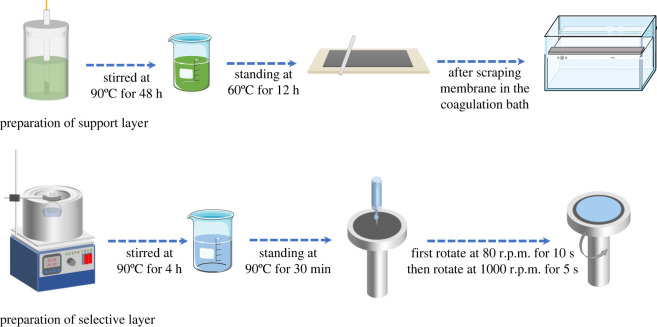
Process for preparing the composite membrane.

To prepare the solution for spin-coating the selective layer, dried PEBA and n-butanol solvent in a mass ratio of 1 : 9 were placed in a conical flask, which was then sealed, and the mixture was stirred continuously for 4 h at 90°C with a magnetic thermostat stirrer to fully dissolve the PEBA. Subsequently, a certain mass of MCM-41 or Silicalite-1 zeolite was added, followed by stirring for 1 h. After being fully mixed, the spin-coating solution obtained was kept in a water bath at 90°C for 30 min to remove bubbles. The support layer was cut into rounds the same size as the spin-coated substrate so that it could fit perfectly on the substrate. The selective layer was prepared by a spin coater using the static drip method, by rotating at a low speed of 80 r.p.m^.^ for 10 s and then at a high speed of 1000 r.p.m^.^ for 5 s. The thus-prepared composite membrane was dried naturally and set aside. The entire preparation flow is shown schematically in figure 1.

Using this method, three different PEBA composite membranes were prepared: one without modification, one modified with MCM-41 and one modified with Silicalite-1.

### 2.3. Membrane characterization methods

The membrane morphology was examined using a field emission scanning electron microscope (SEM; Hitachi S4800, Japan). To prepare the samples, the composite membranes were immersed in liquid nitrogen, and the membrane samples were carefully fractured. After being coated with gold using an ion sputtering device, the surface and cross-sectional morphologies were examined in the SEM. The Fourier transform infrared spectroscopy (FTIR) of the support layer and composite membranes were recorded by employing an infrared spectrometer (Agilent Cary660 + 620, China) to determine whether chemical changes occurred before and after the modification. Water contact angles on the membranes were measured using a contact angle analyser (OCA200, Germany) to examine their hydrophobicity.

### 2.4. Gas permeation test

**Figure 2.**
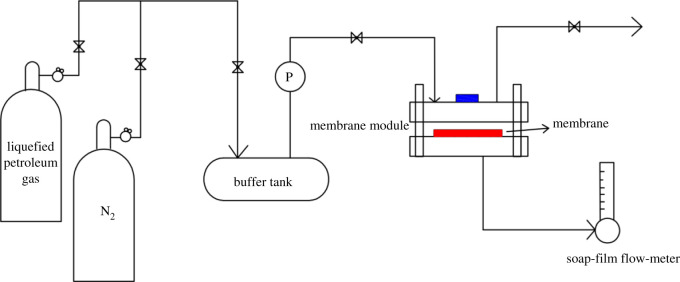
Schematic view of the experimental set-up for removal of organic vapours.

The experimental set-up for the removal of organic vapour from liquified petroleum gas (LPG)/N_2_ mixtures using membranes is shown in figure 2. The main components of LPG include ethane, propane, cyclopropane, n-butane and isobutane. Its composition is essentially the same as that of volatile petroleum gas. In order to determine the gas permeance, a soap-film flow-meter was connected to the residue outlet of the membrane module, and the inlet valve was closed. The permeance *J* (mol m^−2^ s^−1^ Pa^−1^) was then calculated using the following equation:

$$J=\frac{F}{22400}+\frac{273.15}{T}+\frac{1}{A(\;p_{f}+p_{p})},$$

where *F* is the volumetric permeation rate (ml s^−1^) of the measured gas at ambient conditions, *T* is room temperature (K), *A* is the effective membrane area (m^2^), *p_f_* is the upstream (feed) pressure (Pa) and *p*_p_ is the downstream pressure (Pa). The gases before and after separation were collected separately using sample bags, and the gas concentration of each component was detected by a gas chromatograph (Fuli GC9720, China). The column used was KB-Al_2_O_3_/Na_2_SO_4_ (length: 50 m, inner diameter: 0.53 mm). The oven, injector and detector temperatures were set to 120, 100 and 180°C, respectively. The separation performance of each composite membrane was analysed by comparing the gas concentration before and after separation.

## 3. Results and discussion

### 3.1. Membrane characterization

**Figure 3.**
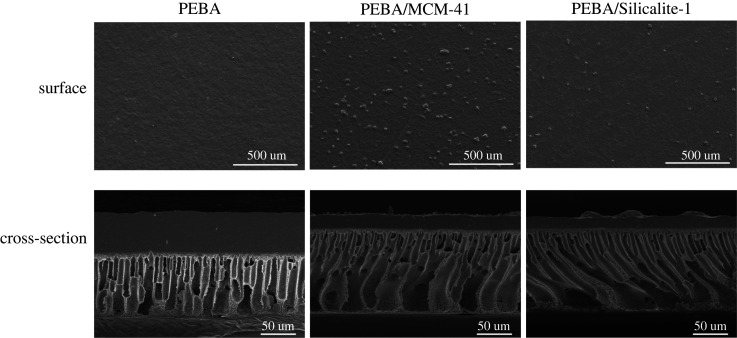
SEM images of PEBA, PEBA/MCM-41 and PEBA/Silicalite-1 membranes. Magnification: 1000×.

Cross-sectional and surface SEM photographs of the PEBA/PES membranes with different zeolites are shown in figure 3. The surface of the pure PEBA membrane appears smooth, whereas the PEBA/MCM-41 and PEBA/Silicalite-1 membranes are rough. This difference is attributed to the elimination of intermolecular hydrogen bonding and a decline in the crystallinity of the polyether and polyamide blocks owing to the addition of MCM-41 and Silicalite-1 to the PEBA matrix. Because MCM-41 is more agglomerated than Silicalite-1, the large MCM-41 particles are more obvious on the membrane surface at the same magnification.

The cross-sectional images of the membrane demonstrate the finger-like pore structure of the support layer. This structure allows the support layer to be well supported without hindering the separation of the gas. The addition of zeolites increases the density of the spin-coating solution, which in turn increases the centrifugal force. When the selective layer is deposited by spin-coating at the same speed, the zeolite-modified coating is thinner than the pure PEBA coating. The thinner coating layer enables organic gases to pass through the membrane with less resistance, thus increasing its gas permeance. In addition, the cross-sectional image of the separation layer on the unmodified membrane shows that it is filled with PEBA polymer and uniformly coated, whereas those of the modified membranes indicate that zeolite particles are completely embedded in the PEBA matrix, and some textures are produced inside the separation layer. The addition of zeolites increases the pore space, interrupts the original polymer connectivity and makes the internal matrix connections discontinuous. Such a pore structure is also conducive to increasing the permeability through the membrane.

**Figure 4.**
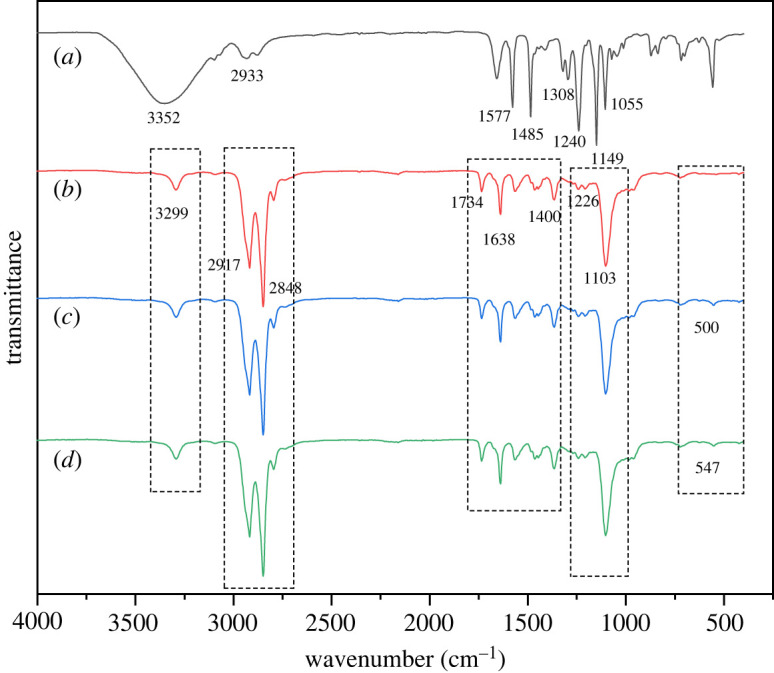
FTIR spectra of the (a) support layer and the (b) PEBA, (c) PEBA/MCM-41 and (d) PEBA/Silicalite-1 membranes.

The FTIR spectra of the support layer and the three composite membranes are shown in figure 4. For the support layer, the strong twin peaks at 1055 and 1240 cm^−1^ are attributed to the symmetric and antisymmetric stretching vibrations of C–O–C, respectively. Meanwhile, the peaks at 1149 and 1308 cm^−1^ are assigned to the symmetric and antisymmetric stretching vibrations of O=S=O, respectively. Those at 1485, 1577 and 2933 cm^−1^ arise from the C=C and C–H bond stretching vibration in benzene rings; in addition, the NMP decompositions in the range of 3000–3500 cm^−1^ appear in a broader hydrogen bonding region. For the pure PEBA composite membrane, the peaks at 3299, 1638 and 1400 cm^−1^ correspond to N–H, C=O and C–H in the imidazole ring, respectively. The imide group is characterized by bands at 1103, 1734, 2917 and 2848 cm^−1^ (asymmetric stretching of C–O–C and C=O in the imide group); 1226 cm^−1^ (symmetric stretch of C–O in the imide group); and 1103 cm^−1^, 1734 cm^−1^, 2917 cm^−1^ and 2848 cm^−1^ (antisymmetric and symmetric stretching vibrational peaks of −CH_2_). The confluent absorption peaks around 500 cm^−1^ could be ascribed to the bending vibrations of tetragonal Si–O–Si bonds from the FTIR spectra of the PEBA/Silicalite-1 composite membrane. The FTIR spectra of the PEBA/Silicalite-1 composite membrane show the characteristic FTIR peak of Silicalite-1 at 547 cm^−1^, which denotes the Si–O–Si structure, specifically, the double-ring tetrahedral vibrations of tetrahedral Si in the zeolite framework, in MFI-structured zeolite of the pure Silicalite-1.

Notably, the two FTIR spectra show different peak positions, demonstrating that the thin coating layer had entirely covered the support layer to form a complete composite membrane. However, the absence of new peaks in the PEBA/MCM-41 and PEBA/Silicalite-1 composite membranes indicates that the two molecular sieves are physically bonded to PEBA and no chemical reaction has occurred. This indicates that there is no chemical reaction between the two molecular sieves and PEBA.

**Figure 5.**
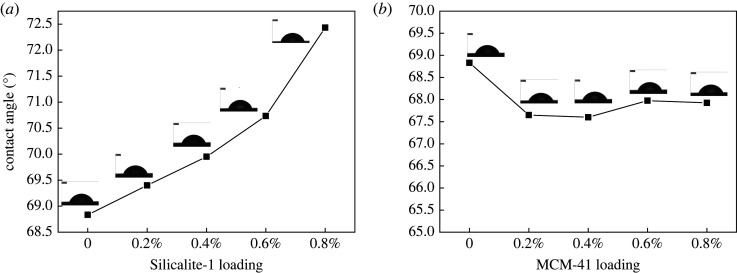
Water contact angles of PEBA/Silicalite-1 (*a*) and PEBA/MCM-41 (*b*) membranes.

The changes in the organic vapour concentration can be explained by the change in the hydrophobicity of the membranes, as illustrated in figure 5. The water contact angle of the Silicalite-1-modified membranes increased from 68° to 72° with the increasing zeolite concentration. With the MCM-41-modified membrane, however, the water contact angle was slightly lower than that of the unmodified membrane, but it remained constant at 68° with the increasing zeolite concentration. Because the skeleton of Silicalite-1 does not contain aluminium ions, it is extremely hydrophobic; thus, it increased the hydrophobicity of the membrane. By contrast, the MCM-41 did not significantly affect the hydrophobicity of the membrane, and only the surface morphology and roughness influenced the contact angle value. The properties of the membrane material dictate the separation performance. Consequently, increasing the membrane hydrophobicity could preferentially absorb more organic compounds, thus increasing the permeate concentration.

### 3.2. Gas separation performance

**Figure 6.**
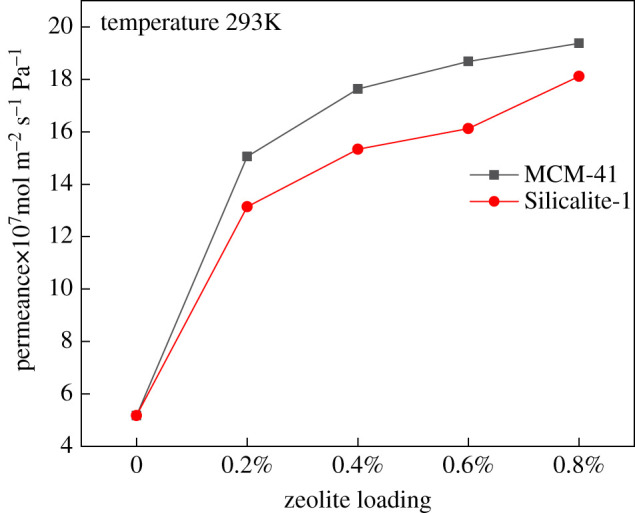
Effect of the zeolite concentration on the membrane permeance.

Ethane, propane, cyclopropane, n-butane and isobutane are the main components of oil vapour. Understanding the separation of these organic vapours is very important in the study of oil vapour removal by composite membranes. The organic gas permeance of zeolite-modified membranes is shown in figure 6, which reveals that increasing the concentration of the added zeolites increased the solubility of the organic vapour. Because permeability is the product of solubility and diffusivity, the permeance increased with the increasing zeolite concentration in the membrane. The gas permeability in these membranes is frequently higher than that of others owing to the excessive diffusional path provided by the pore structure of porous materials and non-selective voids between the silica and polymer particles. Evidently, when the zeolite concentration was sufficiently high, the permeance ratio of organic vapour to N_2_ reached nearly 20, whereas that of the membrane with zeolites was only 4. In addition, the mesoporous structure of MCM-41, which is a molecular sieve, also provides more paths for gases; thus, its permeability is slightly higher than that of Silicalite-1, which is microporous.

**Figure 7.**
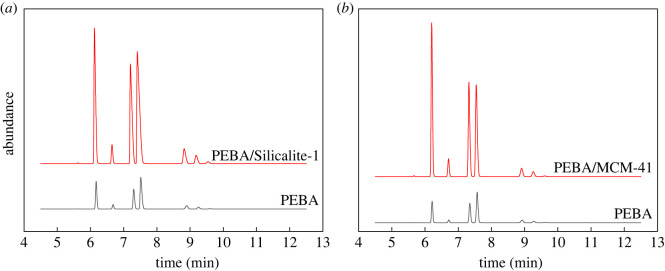
Comparison of the gas chromatography results of the unmodified membranes with that of the Silicalite-1- (*a*) and MCM-41-modified (*b*) membranes.

The effects of the three membranes on the separation of oil vapour are shown in figure 7. The zeolite-modified membranes exhibit an improved ability to separate organic vapours. Without the addition of molecular sieves, the non-selective pores between the polymer do not provide sufficient diffusion paths, so the dissolution–diffusion process dominates. With the increasing content of porous molecular sieves, the number of non-selective pores also increases, and Knudsen diffusion gradually becomes dominant. Adding porous materials can increase the effective adsorption area for organic gas and improve the solubility, thereby increasing the selectivity.

**Figure 8.**
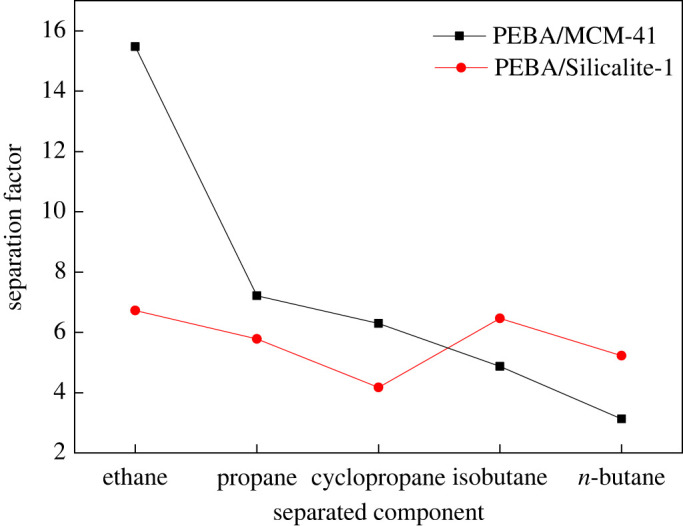
Selectivity of modified membranes normalized to that of unmodified membranes toward each gas component.

Figure 8 shows the separation factor for each of the two modified composite membranes for different gases in the feed stream. The separation factor is a measure of the improvement over the performance of the unmodified membrane (which is already quite effective). Specifically, the selectivity of each modified membrane was normalized to that of the pure PEBA membrane to better compare their selectivities. The MCM-41-modified membrane exhibits a better separation effect for light hydrocarbons; for example, its selectivity for ethane is nearly 16 times higher than that of the unmodified membrane. In addition, the separation rate of the Silicalite-1-modified membrane is more constant across the different gases, but it is approximately 4–7 times higher than that of the unmodified membrane. The separation mechanism differs between the two molecular sieve modifications: dissolution–diffusion dominates in the membrane containing microporous molecular sieves as inorganic fillers, and Knudsen diffusion prevails in that with mesoporous molecular sieves. Normally, gas is transported through the polymer matrix mainly by dissolution–diffusion, which is related to the ability of the gas molecules to penetrate the surface layer of the polymer. Adding mesoporous molecular sieves increases the number of non-selective voids, and the transport mechanism then tends towards Knudsen diffusion. Therefore, the PEBA/Silicalite-1 membrane more effectively separates large molecules. Meanwhile, adding MCM-41 increases the small pores in the membrane matrix, thus facilitating the passage of small molecules through the membrane. In addition, fewer larger pores are added, so the selectivity for large molecules increases by a lower degree. In addition, the complex hexagonal mesoporous structure of MCM-41 hinders the separation of large-molecule gases, so the PEBA/MCM-41 membrane is less efficient in separating heavy hydrocarbons than it is in separating light ones.

## 4. Conclusion

The separation of oil vapour from nitrogen gas using PEBA/MCM-41 and PEBA/Silicalite-1 composite membranes was studied. The following general conclusions can be drawn:

(i) When the microporous Silicalite-1 and mesoporous MCM-41 zeolites are used to modify PEBA composite membranes, the selective layer of the membrane becomes thinner, which increases the gas permeance. Silicalite-1 enhances the hydrophobicity of the membrane and effectively improves its selectivity towards organic vapour.
(ii) Both the PEBA/MCM-41 and PEBA/Silicalite-1 composite membranes can effectively separate oil vapour and show improved permeability and selectivity toward organic gas. Owing to the relatively large pores of MCM-41, the permeability of its corresponding membrane is slightly higher than that of the Silicalite-1-modified membrane. Adding MCM-41 makes the Knudsen diffusion mechanism dominant; thus, it more effectively increases the selectivity for small molecules. By contrast, the dissolution–diffusion mechanism dominates in the membrane modified with Silicalite-1, which evidently improves the ability of PEBA/Silicalite-1 composite membrane to separate large molecules.

In future investigations, we can take advantage of both the high separation capacity of mesoporous molecular sieves for small molecules and the smooth overall separation effect of microporous molecular sieves for gases. It may be possible to combine these two functionalities to investigate new modifications that can both improve the separation efficiency and equalize the separation of the various gas components.

## Data Availability

The Dryad access link is as follows: https://doi.org/10.5061/dryad.8pk0p2nqq [37].

## References

[1] Gonzalez DJ, Francis CK, Shaw GM, Cullen MR, Baiocchi M, Burke M. 2022 Upstream oil and gas production and ambient air pollution in California. Sci. Total Environ. **806**, 150298. (10.1016/J.SCITOTENV.2021.150298) 34844318

[2] MacKay K, Lavoie M, Bourlon E, Atherton E, O'Connell E, Baillie J, Fougère C, Risk D. 2021 Methane emissions from upstream oil and gas production in Canada are underestimated. Sci. Rep. **11**, 8041. (10.1038/S41598-021-87610-3) 33850238 PMC8044210

[3] Liu N, Sun H, Xu L, Cai Y. 2021 Methylsiloxanes in petroleum refinery facility: their sources, emissions, environmental distributions and occupational exposure. Environ. Int. **152**, 106471. (10.1016/J.ENVINT.2021.106471) 33676090

[4] Wilde SE et al. 2021 Speciation of VOC emissions related to offshore North Sea oil and gas production. Atmos. Chem. Phys. **21**, 3741-3762. (10.5194/ACP-21-3741-2021)

[5] Tzompa-Sosa ZA et al. 2019 Atmospheric implications of large C2-C5 alkane emissions from the U.S. oil and gas industry. J. Geophys. Res. Atmos. **124**, 1148-1169. (10.1029/2018JD028955) 32832312 PMC7433792

[6] Franco B et al. 2016 Evaluating ethane and methane emissions associated with the development of oil and natural gas extraction in North America. Environ. Res. Lett. **11**, 044010. (10.1088/1748-9326/11/4/044010)

[7] Dalane K, Dai Z, Mogseth G, Hillestad M, Deng L. 2017 Potential applications of membrane separation for subsea natural gas processing: a review. J. Nat. Gas Sci. Eng. **39**, 101-117. (10.1016/j.jngse.2017.01.023)

[8] Triebe R. 2015 Condensing heat recovery for industrial process applications. Process Heating **22**, 26-30.

[9] Keinath BL, Garimella S. 2018 High-pressure condensing refrigerant flows through microchannels. Part I: pressure drop models. Heat Transf. Eng. **40**, 818-829. (10.1080/01457632.2018.1443257)

[10] Wang Y, Jin Z. 2019 Effect of pore size distribution on hydrocarbon mixtures adsorption in shale nanoporous media from engineering density functional theory. Fuel **254**, 115650. (10.1016/j.fuel.2019.115650)

[11] Shen Y, Shi W, Zhang D, Na P, Tang Z. 2019 Recovery of light hydrocarbons from natural gas by vacuum pressure swing adsorption process. J. Nat. Gas Sci. Eng. **68**, 102895. (10.1016/j.jngse.2019.05.008)

[12] Liemberger W, Gross M, Miltner M, Harasek M. 2017 Experimental analysis of membrane and pressure swing adsorption (PSA) for the hydrogen separation from natural gas. J. Clean. Prod. **167**, 896-907. (10.1016/j.jclepro.2017.08.012)

[13] Ghorbani B, Shirmohammadi R, Mehrpooya M. 2018 A novel energy efficient LNG/NGL recovery process using absorption and mixed refrigerant refrigeration cycles – economic and exergy analyses. Appl. Therm. Eng. **132**, 283-295. (10.1016/j.applthermaleng.2017.12.099)

[14] Ruan X, Wang L, Dai Y, Zhang N, Yan X, He G. 2016 Effective reclamation of vent gas in ethylbenzene dehydrogenation by coupling multi-stage circle absorption and membrane units. Sep. Purif. Technol. **168**, 265-274. (10.1016/j.seppur.2016.05.061)

[15] Pintor AMA, Vilar VJP, Botelho CMS, Boaventura RAR. 2016 Oil and grease removal from wastewaters: sorption treatment as an alternative to state-of-the-art technologies. A critical review. Chem. Eng. J. **297**, 229-255. (10.1016/j.cej.2016.03.121)

[16] Sun C, Wen B, Bai B. 2015 Application of nanoporous graphene membranes in natural gas processing: molecular simulations of CH_4_/CO_2_, CH_4_/H_2_S and CH_4_/N_2_ separation. Chem. Eng. Sci. **138**, 616-621. (10.1016/j.ces.2015.08.049)

[17] Zhou D, Zhong Y, Yang J, Qi J, Zhuo Y, Sha Y. 2018 Preparation and application of PDMS/PES composite membrane in separating light hydrocarbon components from drilling mud. J. Membr. Sci. **566**, 231-238. (10.1016/j.memsci.2018.09.008)

[18] Awad A, Aljundi IH. 2018 Layer-by-layer assembly of carbide derived carbon-polyamide membrane for CO_2_ separation from natural gas. Energy **157**, 188-199. (10.1016/j.energy.2018.05.136)

[19] Eguchi H, Kim DJ, Koros WJ. 2015 Chemically cross-linkable polyimide membranes for improved transport plasticization resistance for natural gas separation. Polymer **58**, 121-129. (10.1016/j.polymer.2014.12.064)

[20] Momeni M, Kojabad ME, Khanmohammadi S, Farhadi Z, Ghalandarzadeh R, Babaluo A, Zare M. 2018 Impact of support on the fabrication of poly (ether-b-amide) composite membrane and economic evaluation for natural gas sweetening. J. Nat. Gas. Sci. Eng. **62**, 236-246. (10.1016/j.jngse.2018.12.014)

[21] Shahid S, Nijmeijer K. 2017 Matrimid®/polysulfone blend mixed matrix membranes containing ZIF-8 nanoparticles for high pressure stability in natural gas separation. Sep. Purif. Technol. **189**, 90-100. (10.1016/j.seppur.2017.07.075)

[22] Hejtmánek V, Schneider P, Soukup K, Šolcová O. 2007 Comparison of transport characteristics and textural properties of porous material; the role of pore sizes and their distributions. Stud. Surf. Sci. Catal. **160**, 217-224. (10.1016/S0167-2991(07)80029-2)

[23] Kim H, Kim Y, Yoon M, Lim PS, Seo G, Kim K. 2010 Highly selective carbon dioxide sorption in an organic molecular porous material. J. Am. Chem. Soc. **132**, 12 200-12 202. (10.1021/ja105211w) 20718409

[24] Maretto M, Vignola R, Williams CD, Bagatin R, Latini A, Papini MP. 2015 Adsorption of hydrocarbons from industrial wastewater onto a silica mesoporous material: structural and thermal study. Microporous Mesoporous Mater. **203**, 139-150. (10.1016/j.micromeso.2014.10.021)

[25] Takahashi M, Fuji M. 2014 Synthesis and fabrication of inorganic porous materials: from nanometer to millimeter sizes. Kona **20**, 84-97. (10.14356/kona.2002011)

[26] Lee DS, Liu TK. 2002 The synthesis and characteristics of vanadoaluminosilicate MCM-41 mesoporous molecular sieves. J. Solgel Sci. Technol. **23**, 15-25. (10.1023/A:1013367602166)

[27] Lupulescu AI, Rimer JD. 2014 In situ imaging of Silicalite-1 surface growth reveals the mechanism of crystallization. Science **344**, 729-732. (10.1126/science.1250984) 24833388

[28] Burmann P, Zornoza B, Téllez C, Coronas J. 2014 Mixed matrix membranes comprising MOFs and porous silicate fillers prepared via spin coating for gas separation. Chem. Eng. Sci. **107**, 66-75. (10.1016/j.ces.2013.12.001)

[29] Weng TH, Tseng HH, Wey MY. 2010 Fabrication and characterization of poly(phenylene oxide)/SBA-15/carbon molecule sieve multilayer mixed matrix membrane for gas separation. Int. J. Hydrogen Energy **35**, 6971-6983. (10.1016/j.ijhydene.2010.04.024)

[30] Kim S, Marand E. 2007 High permeability nano-composite membranes based on mesoporous MCM-41 nanoparticles in a polysulfone matrix. Microporous Mesoporous Mater. **114**, 129-136. (10.1016/j.micromeso.2007.12.028)

[31] Khan AL, Klaysom C, Gahlaut A, Vankelecom IFJ. 2013 Polysulfone acrylate membranes containing functionalized mesoporous MCM-41 for CO_2_ separation. J. Membr. Sci. **436**, 145-153. (10.1016/j.memsci.2013.02.023)

[32] Reid BD, Ruiz-Trevino FA, Musselman IH, Balkus Jr KJ, Ferraris JP. 2001 Gas permeability properties of polysulfone membranes containing the mesoporous molecular sieve MCM-41. Chem. Mater. **13**, 2366-2373. (10.1021/cm000931+)

[33] Bento A, Lourenco JP, Fernandes AJS, Ribeiro MR, Arranzandres J, Lorenzo V, Cerrada M. 2012 Gas permeability properties of decorated MCM-41/polyethylene hybrids prepared by in-situ polymerization. J. Membr. Sci*.* **415–416**, 702-711. (10.1016/j.memsci.2012.05.058)

[34] Campos JM, Lourenço JP, Pérez E, Cerrada ML, Ribeiro MR. 2009 Self-reinforced hybrid polyethylene/MCM-41 nanocomposites: in-situ polymerisation and effect of MCM-41 content on rigidity. J. Nanosci. Nanotechnol. **9**, 3966-3974. (10.1166/jnn.2009.1298) 19504949

[35] Galve A, Sieffert S, Staudt C, Ferrando M, Güell C, Téllez C, Coronas J. 2013 Combination of ordered mesoporous silica MCM-41 and layered titanosilicate JDF-L1 fillers for 6FDA-based copolyimide mixed matrix membranes. J. Membr. Sci. **431**, 163-170. (10.1016/j.memsci.2012.12.046)

[36] Setiawan WK, Chiang KY. 2019 Silica applied as mixed matrix membrane inorganic filler for gas separation: a review. Sustain. Environ. Res. **29**, 1-21. (10.1186/s42834-019-0028-1)

[37] Yan J, Cai Y, Xu R, Wang B, Nie L. 2022 Data from: Separation of oil vapour by polyether block amide composite membrane modified with porous materials. Dryad Digital Repository. (10.5061/dryad.8pk0p2nqq) PMC9682297 36425523

